# The effect of aerobic exercise on bladder function and lower urinary tract symptoms in women who have diabetes mellitus with lower urinary tract symptoms: a randomized controlled

**DOI:** 10.1007/s11845-026-04386-z

**Published:** 2026-04-18

**Authors:** Bengisu Tufekci, Günseli Usgu, Ahmet Tufekci

**Affiliations:** 1 Vocational School of Health Services, Department of Physiotherapy and Rehabilitation, Gaziantep Islam Science and Technology University, Gaziantep, Turkey; 2 Faculty of Health Sciences, Department of Physiotherapy and Rehabilitation, Hasan Kalyoncu University, Gaziantep, Turkey; 3 Faculty of Medicine, Department of Urology, SANKO University, Gaziantep, Turkey

**Keywords:** Lower urinary tract symptoms, Bladder, Diabetes, Aerobic exercise, Quality of life

## Abstract

**Background:**

Chronic hyperglycemia monitored by HbA1c may be associated with bladder dysfunction and lower urinary tract symptoms in women with diabetes mellitus, but the role of aerobic exercise in this process is unclear.

**Aims:**

The aim of our study is to investigate the impact of aerobic exercise (AE) on bladder function and urinary symptoms in women with Diabetes Mellitus (DM) who had Lower Urinary Tract Symptoms (LUTS).

**Methods:**

The study included 44 women randomly assigned to AE (*n* = 22) and Control (*n* = 22) groups. Both received behavioral therapy; the AE group additionally performed moderate-intensity aerobic exercise. Bladder function was assessed by urodynamics, blood glucose by HbA1c, voiding frequency by a 48-hour bladder diary, and urinary symptoms and quality of life by King’s Health Questionnaire (KHQ) and Bristol Female Lower Urinary Tract Symptoms (BFLUTS) questionnaire. Trial registration: ClinicalTrials.gov, NCT06249399.

**Results:**

The groups had similar characteristic urodynamic features before treatment (*p* > 0.05). However after exercise training, improvements in favour of AE group regarding the following urodynamic parameters as voiding time (*p* = 0.003), voided volume (*p* = 0.008), first urge to urinate (*p* = 0.003), compliance (*p* < 0.001), maximum bladder capacity (*p* = 0.021), subheadings of KHQ (general health (*p* = 0.024), social limitation (*p* = 0.013), mood (*p* = 0.001), symptom severity) (*p* = 0.042), and of BFLUTS (storage functions (*p* = 0.004), quality of life (*p* = 0.017), and total scores (*p* = 0.012)), urinary frequency (*p* = 0.003), and blood glucose levels (*p* = 0.002).

**Conclusion:**

AE training for women with DM and LUTS, was associated with improved bladder function, urinary symptoms and urinary system related quality of life.

## Highlights

AE training for women with DM and LUTS, was associated with improved bladder function.

AE training showed a beneficial effect on urinary system symptoms in DM and LUTS.

AE training improved urinary system related QOL in women with DM and LUTS.

## Introduction

Lower urinary tract symptoms (LUTS) has been defined as a series of clinical symptoms indicating bladder storage and voiding dysfunction [1]. Diabetes Mellitus (DM) is a chronic disease characterized by disorders of carbohydrate, fat, and protein metabolism resulting from hyperglycemia caused by absolute or relative insulin deficiency or insulin resistance [2]. LUTS associated with DM is linked to damage and decreased viability in the parasympathetic neurons of the pelvic ganglia due to chronic hyperglycemia [3]. Another reason for the development of LUTS in individuals with DM is thought to be the microvascular and neurological complications caused by hyperglycemia, which lead to developmement of autonomic neuropathy in affected individuals [4]. Studies have reported that in women with diabetes, 8–9 years after diagnosis, especially when HbA1c levels are above 7%, the risk of developing bladder dysfunction increases [5]. LUTS has been shown to be more prevalent and more severe in women with diabetes compared to other groups [6]. Although LUTS does not directly threaten women’s lives, it significantly and negatively affects their daily lives impairing their roles in family and social life. Therefore, the treatment methods applied aiming to improve quality of life carry critical importance [7, 8].

The American College of Obstetricians and Gynecologists (ACOG) (Grade A) and the International Federation of Gynecology and Obstetrics Working Group (FIGO) recommend behavioral and physical therapies (bladder training, exercise, scheduled micturition, reduction in the intake of caffeinated beverages, smoking cessation, prevention of chronic constipation, pelvic floor muscle training, electrical stimulation, biofeedback) among conservative treatments due to the lowest risk of harm in LUTS treatment before resorting to invasive approaches [9–12].

Aerobic exercise (AE) is a type of exercise training that involves rhythmic body movements such as walking, running, and swimming, which rely on working out large muscle groups and utilize aerobic metabolism to obtain energy [13]. Regular aerobic exercise training in patients with diabetes plays an important role in glycemic control by reducing HbA1c levels [14]. Achieving glycemic control reduces nerve fiber loss and the severity of peripheral sensorimotor polyneuropathy in people with diabetes [15]. At the same time, studies have shown that aerobic exercises performed at submaximal levels increase expressions of BDNF (Brain Derived Neurotrophic Factor) and NGF (Nerve Growth Factor) which have a neuroprotective effect against neuron damage in diabetes-related peripheral neuropathy [16–18]. Considering the etiopathology of diabetes-related LUTS, it is important to note that AE may be effective in managing LUTS problems.

There are studies in the literature investigating the effectiveness of conservative treatments such as training of pelvic floor muscle and behavioral treatment approaches for LUTS [19]. However, the effect of aerobic exercise on bladder function and urinary system symptoms in women with DM and LUTS has not been investigated so far.

The aim of our study is to investigate the effectiveness of AE training, administered in addition to routine behavioral modification therapy, on bladder function, urinary system symptoms, and quality of life in women with DM who presented to the clinic with LUTS.

## Methods

For this study, permission was obtained from the Hasan Kalyoncu University Health Sciences Non-Interventional Research Ethics Committee on April 27, 2023 (No: 2023/43). The study was conducted prospectively between May 2023 and October 2024 on female patients who applied to the Urology Outpatient Clinic of XXX Research and Practice Hospital with LUTS but without previous diagnosis of diabetes and any suspicion of pregnancy at the time of presentation. All study participants who gave their written informed consent were enrolled in the study (Clinical Trials No: NCT06249399).

**Fig. 1:**
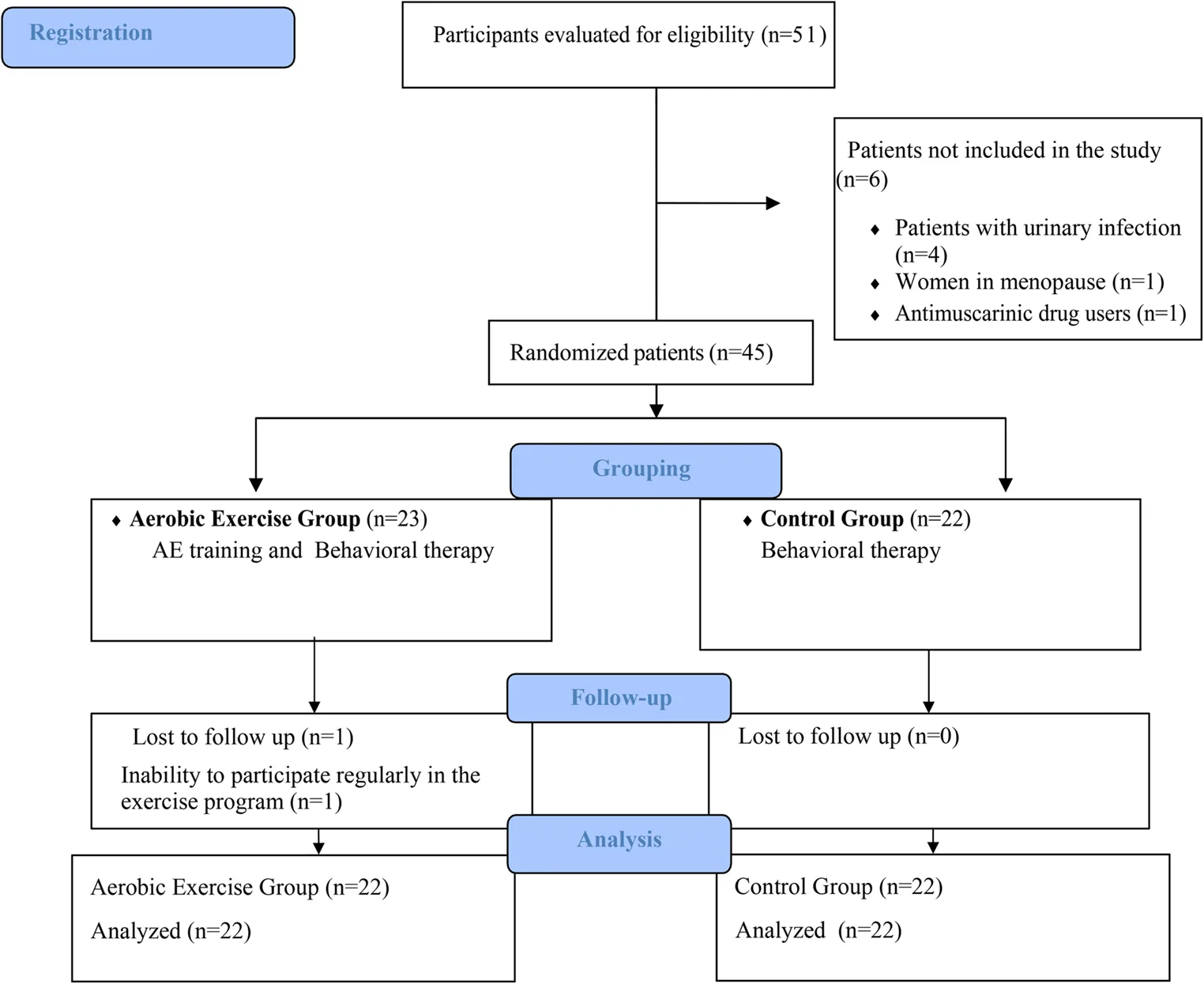
Flow diagram of the research study

As the primary outcome measures for treatment efficacy, in consideration of the results of a study by Tong Y. et al. on diabetic bladder dysfunction, urodynamic assessments specifically bladder sensation (first urge to urinate) and compliance were referenced [20]. To determine the sample size, a power analysis (G*power sample size calculator) was performed with a 5% Type-1 and 20% Type-2 error margins. With an effect size of 0.90, 22 patients were included in each group, and the total number of 44 participants consisted the study population. The study population included female patients aged 35–55 years with a diagnosis of LUTS and had Type II diabetes for 5 years or more. Patients with stage 2 or higher pelvic organ prolapse or received the diagnoses of urinary tract infection, cerebrovascular disease, or dementia; users of alpha blockers, antimuscarinics, antidepressants, diuretics, and antihistamines; women who had previously undergone surgery for urinary incontinence and pelvic organ prolapse; and those in menopause were excluded from the study. Participants were randomized to either the Aerobic Exercise (AE) group (*n* = 22) or the Control group (*n* = 22) using a simple randomization method implemented with sealed envelopes. To ensure allocation concealment, the envelopes were prepared by an independent physiotherapist researcher who was not involved in delivering the intervention. After baseline assessment, participants were assigned to groups according to the sealed envelopes. No changes in medical treatments related to diabetes or bladder symptoms were reported during follow-up. The study flow diagram is shown in Fig. 1.

### Assessments

A patient information form prepared by us was used to determine the participants’ physical (age, height, weight), demographic (marital status, education, smoking/alcohol use), and clinical characteristics (chronic disease, medication use, birth history, surgical procedures), as well as their bladder habits and urination frequency. In the study, participants’ urodynamic and uroflowmetry values were recorded as the primary assessment method for voiding and bladder function. Routine urine analysis performed at the urology outpatient clinic where the study was conducted recorded the presence of glucose and infection in the urine, and blood glucose level monitoring recorded HbA1c level values. Bladder wall thickness monitoring was performed at the urology outpatient clinic where the study was conducted, and bladder ultrasound values were obtained. A 48-hour voiding diary was used to monitor voiding frequency. The severity of urinary system symptoms was assessed using the King’s Health Questionnaire (KHQ), and the quality of life related to urinary system problems was assessed using the Bristol Female Lower Urinary Tract Symptoms (BFLUTS) questionnaire. All assessments were performed at baseline before the initiation of the 12-week intervention and repeated after each participant completed the 12-week treatment period in both groups.

#### Bladder functions

##### Urodynamics

Urodynamic assessments were performed using the MMS-Solar Blue urodynamic system (MMS, The Netherlands) in the Urology Outpatient Clinic of XXX Research and Practice Hospital by a clinician blinded to group allocation. The examination was conducted in accordance with the terminology and principles of Good Urodynamic Practice recommended by the International Continence Society (ICS). Filling cystometry was performed using sterile isotonic saline at a filling rate of 50 mL/min according to a standardized institutional protocol, with the participant in the sitting position. Vesical and abdominal pressures were recorded using a catheter-based pressure measurement system, including a 6–7 Fr transurethral catheter, in accordance with standard urodynamic practice. During the filling phase, bladder sensation, first urge to void, cystometric bladder capacity, detrusor activity, and bladder compliance were recorded. Bladder compliance was defined as the change in bladder volume divided by the change in detrusor pressure during filling (ΔV/ΔPdet). Pressure-flow parameters obtained during the voiding phase were also recorded and analyzed [21].

##### Ultrasonography (USG)

Ultrasonography (USG) was performed by the physician, taking the thickest measured part of the bladder wall as a reference using Acuson S2000 ultrasound device ( Siemens AG, Berlin, Germany) so as to evaluate bladder wall thickness. The USG evaluation results of the participants were obtained from the Urology Outpatient Clinic of XXX Research and Practice Hospital [22].

##### Urination frequency

Bladder diary data were collected using a 48-hour bladder diary forms completed over two consecutive days. The averages of daily urination frequencies and amounts of daily fluid intake were taken into account [23]. Participants were instructed to maintain their usual fluid intake and daily habits during diary completion. If diary entries were incomplete, participants were asked to complete the missing record before analysis.

##### Urinary system symptom severity

The KHQ is a measure that determines the impact of urinary system symptoms on individuals’ quality of life. The scale has been found to have high internal consistency (Cronbach’s alpha ≥ 0.68) and test-retest reliability. The KHQ scale consists of two sections and 32 items that examine general health and quality of life and the presence and severity of lower urinary tract symptoms. Scoring on the Symptom Severity Scale ranges from 0 points (best) to 30 points (worst). In other KHQ subscales, the scale scoring ranges from 0 points (best) to 100 points (worst). An increase in the score obtained using KHQ scale indicates a decrease in quality of life [24].

#### Quality of life related to urinary system symptoms

The Bristol Female Lower Urinary Tract Symptoms (BFLUTS) scale was used to assess quality of life related to lower urinary tract symptoms. BFLUTS evaluates the effect of urinary incontinence and LUTS on sexual function and quality of life. The maximum score that can be obtained from the questionnaire is 71, while the minimum score is 0. A high score indicates severe LUTS and a negative impact on sexual function and quality of life [25].

#### Aerobic exercise capacity

The Incremental Shuttle Walk Test (ISWT) was used to determine exercise capacity. The ISWT is a test in which the subject walks between two cones placed 10 m apart at a gradually increasing speed for 12 min, with each 10 m counted as one shuttle. The assessment is based on the measured walking distance. Healthy individuals aged 40–49 are expected to cover 824 m, those aged 50–59 are expected to cover 788 m, those aged 60–69 are expected to cover 699 m, and those aged 70 and above are expected to cover 633 m [26].

### Interventions

#### Behavioral therapy

Participants in the AE and control group received behavioral therapy provided by a physiotherapist trained in pelvic floor rehabilitation for 12 weeks. Behavioral therapy was applied, including parameters such as restriction of fluid intake, regulation of the gastrointestinal system, and avoidance of irritant foods (11). In the behavioral therapy approach, the treatment process consisted of an initial consultation (40 min) and three follow-up consultations (20 min) once a month. All individuals included in the study received behavioral therapy training individually.

#### Aerobic exercise

Aerobic exercise training was conducted by the same physiotherapist via video conference method for 12 weeks, three days a week, for 45 min a day (10 min of warm-up exercise, 30 min of aerobic exercise, and five minutes of cool-down exercise). Aerobic exercise training sessions were conducted with a maximum of three individuals. A metronome was used to ensure that individuals maintained rhythm during aerobic exercise [27]. Aerobic exercise training is described in Table 4. Exercise training was supported by providing an information form containing rules that patients should pay attention to and comply with in order to prevent complications associated with DM that may occur before, during, and after aerobic exercise. BORG Rating of Perceived Exertion (RPE) scale was used to determine the intensity of the exercises and limit symptoms. Moderate-intensity exercise training was provided, with the perceived fatigue level according to RPE scale scores increased from 11 to 12 to 13–14 [28]. Before each exercise session, patients were asked to use their own glucometers to check their blood glucose levels. No serious adverse events, including hypoglycemia, falls, or musculoskeletal complaints, requiring medical intervention or discontinuation were reported during the study period. To increase participants’ adherence to the exercise program, in addition to face-to-face individualized AE training, videos of the exercise types included in our AE program obtained from the YouTube platform were edited by the researchers and provided to the participants. Thus participants were able to make preparations for the video conference sessions in advance. Participants in the control group were instructed not to initiate any new exercise program during the study period, and this was checked during the monthly follow-up consultations.

### Statistical analysis

IBM-SPSS 25.0 (Version 25, Chicago, USA) was used for the statistical analysis of the study findings. In presenting the descriptive data included in the study, arithmetic mean ± standard deviation (X ± SD), minimum (min), and maximum (max) values were used for numerical variables, while numbers (n) and percentages (%) for categorical variables. The normal distribution analysis of the obtained data was performed using the Kolmogorov-Smirnov Test. Comparisons between groups were performed using the Mann-Whitney U Test for data that did not show a normal distribution and the Independent t-Test for data with normal distribution. The chi-square test was used for comparisons made for categorical variables. The Wilcoxon Test was used for intra-group comparisons of pre- and post-treatment values of the data. In all analyses performed, the level of statistical significance was accepted as *p* < 0.05. The effect size of the evaluated data was calculated using the Cohen’s d coefficient. The obtained Cohen’s coefficient was considered small if it was less than 0.20, medium if it was 0.50, and strong if it was 0.80 [29].

## Results

At the start of the study, 51 participants were evaluated, and 6 participants who did not meet the inclusion criteria were excluded from the study. Forty-five women diagnosed with LUTS were included in the study and randomly assigned to AE (*n* = 23), and control (*n* = 22) groups. One participant from the AE group was excluded from the study because she did not regularly participate in the exercise program, and the study was completed with 44 participants (Fig. 1).

There were no differences between the groups in terms of clinical and demographic characteristics at baseline assessment (*p* > 0.05) (Table 1).

**Table 1:** Clinical and demographic characteristics of the study participants

Characteristic features	AE Group (*n* = 22)	Control Group (*n* = 22)	X^2^	*P* ^a^
	*n* %	*n* %		
Constipation Yes No	7 31.8 15 68.2	5 22.7 17 77.3	0.458	0.498
Number of births Never given birth 1–2 births ≥3 births	4 18.2 6 27.3 12 54.5	2 9.1 5 22.7 15 68.2	1.091	0.580
Types of delivery Vaginal birth C-section Both	13 59.1 2 9.1 3 13.6	14 63.6 3 13.6 3 13.6	0.904	0.825
Physical characteristics	**X ± SD Min-Max**	**X ± SD Min-Max**	**t**	**P** ^**a**^
Age (years)	49 ± 4.55 38–54	50.1 ± 4.3 39–55	-0.852	0.399
Height (cm)	1.6 ± 0.0 1.5–1.7	1.6 ± 0.1 1.5–1.7	-0.163	0.872
Weight (kg) Pre-treatment Post-treatment	73.3 ± 6.4 61–85 71.4 ± 6.3 59–82	70.4 ± 7.1 56–82 70.7 ± 7.2 57–83	1.396 0.334	0.170 0.740
BMI (kg/m^2^) Pre-treatment Post-treatment	28.7 ± 3.3 21.9–36.8 28 ± 3.2 21.2–35.5	27.6 ± 4.3 20.8–34.2 27.8 ± 4.4 21.2–34.2	0.930 0.183	0.358 0.856
p^b^	**<0.001**	0.079		
Duration of DM (years)	9.5 ± 2.3 5–15	10 ± 3.2 5–20	-0.481	0.633

*AE* aerobic exercise, *n*number of participants, X^2^Chi-square test, *DM* Diabetes mellitus, *BMI *Body Mass Index, *X* mean, *SD* standard deviation, *Min* minimum, *Max* maximum;; p^a^:t test; p^b^: paired t test; *p* < 0.05

At the initial assessment, there was a difference between the groups in terms of the residual urine volume (*p* = 0.017), while other urodynamic parameters, USG, HbA1c, and ISWT values were similar between both groups (*p* > 0.05). After exercise training, no differences were found between the groups regarding urodynamic parameters of maximum flow rate, maximum detrusor pressure, and residual urine volume (*p* > 0.05**).** It was determined that the AE group had better values in other urodynamic parameters compared to the control group (*p* > 0.05). No difference was found between the groups with respect to USG values after exercise (*p* > 0.05). HbA1c and ISWT values obtained after exercise were better in the AE group compared to the control group (*p* < 0.05). In intra-group comparisons, improvement in all urodynamic parameters (*p* < 0.05), decrease in both bladder wall thickness (*p* < 0.05), and in HbA1c level (*p* < 0.05), and increase in ISWT values (*p* < 0.05) were observed in the AE group after exercise training. In the control group, improvement was observed in all parameters except maximum bladder capacity (*p* < 0.05), while a decrease in bladder wall thickness and HbA1c levels (*p* < 0.05), without any change in aerobic capacity were observed (*p* > 0.05) (Table 2).

**Table 2:** Urodynamic parameters, USG results, HbA1c, and ISWT values of the participants

		AE Group (*n* = 22)		Control Group (*n* = 22)		t	*P* ^b^
		X ± SD	Min-Max	X ± SD	Min-Max		
Urodynamic parameters							
Maximum Flow Rate (ml/s)	PrT	14.2 ± 4.55	8–23	15.2 **±** 3.8	7–23	-0.795	0.431
	PsT	16.8 ± 4.0	10–24	16.5 **±** 3.9	11–26	0.231	0.818
	P^a^-d	**< 0.001-**1.3		**< 0.001-**1.0			
Duration of Voiding (s)	PrT	25.0 **±** 5.7	15–35	26.8 **±** 4.2	22–37	-1.213	0.232
	PsT	20.82 ± 4.45	13–29	25.0 **±** 4.16	21–34	**-3.187**	**0.003**
	P^a^-d	**< 0.001**-1.4		**0.001**-0.8			
Voided Volume (ml)	PrT	368.9 **±** 74.7	195–480	330.0 **±** 82.8	180–480	1.635	0.110
	PsT	407.1 ± 66.2	280–500	342.7 ± 85.4	185–485	**2.791**	**0.008**
	P^a^-d	**< 0.001**-1.5		**< 0.001**-1.0			
First urge to void (ml)	PrT	98.2 ± 26.3	30–140	108.7 **±** 35.3	60–220	-1.115	0.271
	PsT	143.4 ± 28.1	70–220	114.3 **±** 33.1	75–200	**3.141**	**0.003**
	P^a^-d	**< 0.001**-3.2		**0.019**-0.5			
Maximum Detrusor Pressure (cmH_2_O)	PrT	26.3 ± 3.8	10–24	26.9 **±** 4.6	11–26	-0.431	0.669
	PsT	23.4 **±** 2.7	10–21	25.6 **±** 4.2	10–24	-2.001	0.052
	P^a^-d	**< 0.001**-1.3		**< 0.001**-1.0			
Compliance (ml/cmH_2_O)	PrT	22.3 **±** 6.7	8–35	21.0 **±** 8.2	10–41	0.583	0.563
	PsT	30.8 **±** 6.2	19–42	22.4 **±** 7.5	12–39	**4.063**	**< 0**,**001**
	P^a^-d	**< 0.001**-3.8		**0.003**-0.7			
Maximum Bladder Capacity (ml)	PrT	432.7 **±** 73.9	250–550	397.7 **±** 89.9	225–500	1.411	0.166
	PsT	448.0 **±** 65.0	310–550	351.6 **±** 176.2	0-500	**2.407**	**0.021**
	P^a^-d	**0.001**-0.8		0.067-0.4			
Residual Urine (cc)	PrT	62.5 **±** 37.1	10–130	118.0 **±** 97.7	20–335	**-2.489**	**0.017**
	PsT	39.1 **±** 30.2	0-100	58.9 **±** 50.9	0-190	-1.566	0.125
	P^a^-d	**< 0.001**-1.2		**0.018**-0.5			
USG							
Bladder Wall Thickness of full bladder (mm)	PrT	3.1 **±** 1.3	1–6	3.2 **±** 1.3	1–6	-0.350	0.728
	PsT	2.6 **±** 1.1	1–5	2.6 **±** 1.1	1–5	0.000	1.000
	P^a^-d	**0.009**-0.6		**< 0.001**-0.9			
Bladder Wall Thickness of empty bladder (mm)	PrT	5.7 ± 1.6	3–9	5.3 **±** 1.4	3–9	0.911	0,367
	PsT	4.4 ± 1.3	2–7	4.3 **±** 1.2	2–7	0.238	0,813
	P^a^-d	**< 0.001**-1.4		**< 0.001**-1.2			
HbA1c							
	PrT	8.2 ± 0.8	7.1–9.9	7.9 ± 0.9	6.4–10.1	1.259	0.215
	PsT	6.9 ± 0.6	6.0-7.9	7.7 ± 1.0	6.4–10.5	-3.389	**0.002**
	P^a^-d	**< 0.001**-2.4		**0.016**-0.6			
ISWT (m)		**X ± SD**		**X ± SD**			
	PrT	574.7 ± 103.7		533.5 ± 116.7		1.240	0.222
	PsT	613.6 ± 101.6		532.8 ± 113.8		2.483	**0.017**
	P^a^-d	**< 0.001**-2.9		0.785-0.1			

*n* number of study participants, *AE* aerobic exercise, *USG* ultrasound, *HbA1c* Glycosylated hemoglobin, *ISWT* Incremental Shuttle Walk Test, *X *ortalama, *SD* standard deviation, *Min* minimum, *Max *maksimum, *ml *mililitre, *s *saniye, cmH_2_O centimeter water, *cc *cubic centimeter, *mm* milimetre, *m *metre, *kg* kilogram, *PrT* Pre-treatment, *PsT* Post-treatment, *t *t test, *d* Cohen d, effect size; *p* < 0.05

Bladder diary analysis showed that post-treatment daily voiding frequency was significantly lower in the AE group than in the control group (median 9 (5-12) vs. 10.5 (8-12), *p* = 0.003), whereas no significant difference was observed between the groups at baseline (*p* = 0.310).

Before treatment, the groups were similar regarding all parameters of KHQ and BFLUTS (*p* > 0.05). After aerobic exercise training, improvement was observed in all KHQ parameters in the AE group (*p* < 0.05). No statistical difference was found in the voiding function and sexual life parameters of the BFLUTS scale after AE (*p* > 0.05), while improvements were observed in other parameters (*p* < 0.05). In the control group, no changes were detected in the subparameters of KHQ, namely general health, social limitations, mood, and symptom severity (*p* > 0.05), while improvement was observed in other parameters (*p* < 0.05). Improvement was detected in the total BFLUTS score (*p* < 0.05), while no change was observed in other parameters (*p* > 0.05) (Table 3).

**Table 3:** KHQ ile BFLUTS scores of the study participants

KHQ		AE Group (*n* = 22)		Control Group (*n* = 22)			
		X ± SD	Min-Max	X ± SD	Min-Max	z	*p* ^b^
General Health	PrT	52.3 ± 23.0	25–100	52.3 ± 15.3	25–75	-0.285	0.776
	PsT	35.2 ± 19.9	0–75	48.7 ± 18.1	25–75	-2.252	**0.024**
	p^a^-d	**< 0.001**–1.43		0.083–0.39			
Impact of Incontinence	PrT	60.6 ± 22.2	33.3–100	59.1 ± 17.6	33.3–100	-0.150	0.881
	PsT	40.9 ± 22.8	0-7.6.6	53.0 ± 19.7	33.3–100	-1.642	0.101
	P^a^-d	**0.001**–1.17		**0.046**–0.46			
Role Limitation	PrT	37.8 ± 18.7	16.3–66.7	34.9 ± 14.5	16.7–66.7	-0.292	0.770
	PsT	22.7 ± 19.6	0–50	22.7 ± 18.2	0–50	-0.024	0.981
	P^a^-d	**< 0.001**–2.13		**0.001**–1.15			
Physical Limitation	PrT	35.6 ± 17.3	16.3–66.7	35.6 ± 13.9	16.7–66.7	-0.451	0.652
	PsT	22.0 ± 19.5	0–50	20.5 ± 16.2	0–50	-0.170	0.865
	P^a^-d	**0.001**–1.39		**< 0.001**–1.73			
Social Limitation	PrT	45.0 ± 13.1	22.2–66.7	46.0 ± 12.5	22.2–66.7	-0.267	0.789
	PsT	33.3 ± 14.6	0-55.6	45.0 ± 13.5	22.2–66.7	-2.480	**0.013**
	P^a^-d	**< 0.001**–1.82		0.480 − 0.15			
Personal Relationships	PrT	39.4 ± 16.7	16.3–66.7	35.6 ± 13.9	16.7–66.7	-0.586	0.558
	PsT	24.2 ± 13.4	0–50	26.5 ± 15.1	0–50	-0.722	0.470
	P^a^-d	**< 0.001**–1.49		**0.012**–0.74			
Emotional State	PrT	47.0 ± 14.5	11.1–66.7	46.0 ± 13.4	22.2–66.7	-0.328	0.743
	PsT	28.8 ± 15.2	0-55.6	46.0 ± 15.1	22.2–66.7	-3.206	**0.001**
	P^a^-d	**< 0.001**–2.82		1.000–0.00			
Sleep and Energy Level	PrT	36.3 ± 19.0	0-66.7	27.3 ± 17.5	0–50	-1.214	0.225
	PsT	22.7 ± 18.2	0–50	17.4 ± 17.4	0–50	-0.977	0.329
	P^a^-d	**< 0.001**–2.07		**0.003**–0.89			
Symptom Severity	PrT	52.7 ± 15.7	25–75	50.8 ± 13.6	25–75	-0.674	0.501
	PsT	36.7 ± 17.6	0-66.7	47.7 ± 14.4	25–75	-2.034	**0.042**
	P^a^-d	**< 0.001**-1.50		0.059–0.46			
BFLUTS							
Storage Functions	PrT	5.2 ± 1.4	3–8	5.1 ± 1.3	3–8	-0.354	0.723
	PsT	3.8 ± 1.1	2–6	4.9 ± 1.2	3–8	-2.886	**0.004**
	P^a^-d	**< 0.001**–1.51		0.285 − 0.23			
Voiding Functions	PrT	1.0 ± 1.1	0–3	1.5 ± 1.3	0–4	-1.195	0.232
	PsT	0.7 ± 0.1	0–3	1.0 ± 1.1	0–3	-1.025	0.305
	P^a^-d	0.071–0.32		0.075–0.43			
Incontinence	PrT	2.7 ± 1.7	0–5	2.1 ± 1.7	0–5	-1.364	0.173
	PsT	2.3 ± 1.6	0–5	1.9 ± 1.5	0–4	-0.970	0.332
	P^a^-d	**0.007**–0.69		0.102–0.36			
Sexual Life	PrT	1.3 ± 1.2	0–3	2.0 ± 1.5	0–4	-1.571	0.116
	PsT	1.2 ± 1.1	0–3	1.7 ± 1.5	0–4	-1.213	0.225
	P^a^-d	0.317 − 0.21		0.059–0.43			
Quality of Life	PrT	4.4 ± 1.7	2–8	4.6 ± 1.5	3–9	-0.398	0.690
	PsT	3.1 ± 1.4	1–5	4.2 ± 1.3	2–7	-2.390	**0.017**
	P^a^-d	**< 0.001**–1.44		0.070–0.41			
Total Score	PrT	14.5 ± 4.2	7–22	15.1 ± 3.9	7–24	-0.436	0.663
	PsT	11.1 ± 3.1	6–16	13.7 ± 2.9	7–20	-2.517	**0.012**
	P^a^-d	**< 0.001**–1.71		**0.002**–0.79			

KHQ: King’s Health Questionnaire; BFLUTS: Bristol Female Lower Urinary Tract Symptoms; n:number of participants; AE: aerobic exercise; X:mean; SD: standard deviation; Min: minimum; Max: maksimum; PreT: Pre-treatment; PsT: Post-treatment; z:Mann- Whitney U testi; P^a^:Wilcoxon Test; d: Effect size; *p* < 0.05

## Discussion

The aim of our study is to investigate the effectiveness of aerobic exercise training, applied in addition to behavioral modification therapy, on bladder function, urinary system symptoms, and quality of life in women with diabetes with LUTS. Aerobic exercise training is found to improve bladder function, reduce symptom severity, and improve quality of life in women with LUTS, and aerobic exercise have a higher effect size compared to behavioral therapy Table 4.

**Table 4:** Aerobic exercise training program

Number of repeitions (single set)	1. wk	2. wk		3. wk	4. wk	5. wk	6. wk	7. wk		8. wk	9. wk	10. wk		11. wk	12. wk
	8–10 repetitions					12–15 repetitions					15–20 repetitions				
WARM UP EXERCISES	• Arm rotation • Arm crossover • Scapular adduction exercises • Downward punches • High knee lift		• Side stepping exercise • Torso Twists • Trunk rotation • Swimming exercise • •Jogging in place (Borg 10)			• Arm crossover • Scapular adduction exercises • Butt Kicks • Downward Punches • Side stepping			• High knee lift Trunk rotation • Body rotation • Snow Angels •Jogging in place (Borg 10)		• Arm crossover • Scapular adduction exercises • Butt Kicks • Downward Punches • Side stepping		• High knee lift • Trunk rotation • Body rotation • Side Lunge Windmill •Jogging in place(Borg 10)		
AEROBIC EXERCISES	• Faces • Table Tab • Side Tab Crunch (table Table 2) • Squatting • Slow Jumping Jacks • Slow Star Jumps •Fly Squat		• Jumping Jacks • Ski Jacks • Backstroke • Jumping oblique twists •Jogging in place (Borg 14)			• Faces • Table tab • Hands on knees (table tab) • Squatting • Slow Jumping Jacks • Slow Star Jumps • Fly Squat			• Jumping Jacks • Ski Jacks • Arm reach move • Burpee Side Taps • Backstroke • Jumping oblique twists •Jogging in place (Borg 14)		• Faces • Hands on knees (table tabda) • Squat jacks • Ski Jacks • Ski Jump • Mountain Climber		• Arm reach move • Burpee Side Taps • Floor taps • Backstroke • Jumping oblique twists • •Jogging in place(Borg 14)		
COOL-DOWN EXERCISES	• Side stepping • Torso Twists • Trunk rotation • Body rotation •Thigh Stretch Left		• Thigh Stretch Right • Side stretches • Bridge exercises • Cat -camel exercise • Lying on back with knees bent to the stomach			• Side stepping • Torso Twists • Trunk rotation • Body rotation • Thigh Stretch Left/ Right			• Thigh Stretch Right • Side stretches • Bridge exercises • Cat -camel exercise • Lying on back with knees bent to the stomach		• Side stepping • Torso Twists • Trunk rotation • Body rotation • Thigh Stretch Left/ Right		• Side stretches • Bridge exercises • Cat -camel exercise • Lying on back with knees bent to the stomach • Flank press back		

*wk* week

### Urodynamic and ultrasonographic assessments

While diabetic cystopathy is one of the pathophysiological causes of LUTS in individuals with diabetes, the storage and voiding symptoms observed in these patients vary. The heterogeneity of symptoms seen in diabetic cystopathy is associated with the amount of morphological changes that will occur in the bladder, depending on factors such as the duration of DM, the level of hyperglycemia, and peripheral neuropathy developed as a result of diabetes [20]. Diabetic cystopathy has been reported to cause detrusor hyperactivity in the early stages and detrusor hypoactivity in the advanced stages of bladder muscle tone [30]. In a study conducted in 2018, Wang et al. examined the effects of low-intensity extracorporeal shock wave therapy (Li-ESWT) on bladder function in mice with a diabetes model created by streptozotocin administration. They found that Li-ESWT significantly reduced post-residual volume [31]. Similarly, in our study, we observed that the residual urine volume in the AE group decreased from 62.5 ± 37.1 cc before treatment to 39.1 ± 30.2 cc after treatment. When comparing the treatment efficacy within the groups, we observed that the residual urine volume decreased in both the AE group and the control group, indicating a significant improvement in bladder function in terms of this parameter. However, the treatment protocol applied in the AE group had a high treatment effect size on residual volume, while the control group had a moderate treatment effect size. These findings demonstrate that aerobic exercise has the potential to improve regulation of bladder functions.

We believe that the improvements observed in the AE group in urodynamic parameters, including voiding time, voiding volume, sensation, compliance, and maximum bladder capacity, were due to enhanced glycemic control achieved through aerobic exercise training. The improvement we achieved in the glycemic profile is thought to be effective on peripheral neuropathy associated with diabetes and endothelial and neuronal cell damage in the bladder. This effect is predicted to stem from an increase in BDNF and NGF levels, which have damage-reversing or progression-preventing properties. This biological mechanism may explain the improvement we observed in the five parameters of urodynamics [17, 32, 33]. We believe that another reason may be that the reduction in the severity of hyperglycemia as a result of AE may have led to a decrease in the increased oxidative stress level in bladder muscle cells secondary to DM, thereby healing the resultant endothelial and microvascular damage [34]. The improvement observed in HbA1c values in the AE group also reveals that the planned AE training plays an effective role in glycemic control.

We believe that the reason for the lack of change in maximum flow rate values after aerobic exercise training and behavioral therapy is that the average maximum flow rate values for participants in both groups were within their normal physiological range prior to treatment [35].

In our study, bladder wall thickness was measured using ultrasound to evaluate the morphological structure of the bladder. Previous studies have reported that a bladder wall thickness greater than 5 mm when the bladder is empty may be associated with detrusor dysfunction [36, 37]. In our study, we determined that the wall thickness of the empty bladder was greater than 5 mm in both groups (5.68 ± 1.59 mm in the AE group and 5.27 ± 1.39 mm in the control group). These findings suggest the presence of detrusor dysfunction in the individuals with diabetes included in our study.

Uzun et al. found a significant relationship between overactive bladder (OAB) and bladder wall thickness in persons with and without diabetes. They reported an increase in bladder wall thickness in individuals with DM, which could affect detrusor activity and lead to LUTS [38]. In their studies using rats with diabetes, Wang et al., examined structural changes in the bladder wall and reported that increased bladder wall thickness in DM decreased after application of Li-ESWT, and muscle content increased (31). The finding that wall thickness of empty bladder decreased in both groups is consistent with the relevant literature data. Although bladder wall thickness decreased in both groups after the applications of Li-ESWT, we observed that the treatment effect size was superior in the AE group compared to the control group.

### Lower urinary tract symptom severity and quality of life

LUTS is a common health problem that negatively affects women’s quality of life [39]. Diabetes often accelerates and aggravates the severity of urological complications and LUTS problems that develop with these complications [40]. Findings such as nocturia (polyuria at night), increased residual urine volume, OAB, and urinary incontinence in women with DM explains the higher prevalence of LUTS in this patient population [41].

Celenay and colleagues used the KHQ to assess the quality of life related to OAB symptoms in a study involving 33 women diagnosed with OAB. The areas most affecting the quality of life of participants with OAB were determined according to the KHQ subheadings. The top three areas were reported to be the impact of incontinence, role limitations, and general health perception, while personal relationships were reported to be the least affected area [42]. Our study results are consistent with the literature; according to the KHQ, the top three subheadings with the highest symptom severity in both groups were impact of incontinence, symptom severity, and general health status.

In our study, aerobic exercise sessions were conducted via teleconference, while behavioral therapy follow-up was implemented using a telehealth method. Systematic reviews and meta-analyses in the literature have demonstrated that telehealth-based approaches improve quality of life in women with LUTS [43, 44]. Wadensten et al. reported in their randomized controlled trial involving 123 women that pelvic floor muscle exercises added to behavioral therapy via a mobile application resulted in a significant improvement in LUTS-related quality of life compared to behavioral therapy alone [45].

In a study by Kamalı et al. investigating the effect of e-pelvic floor muscle training (e-PFMT) on the quality of life of women with stress urinary incontinence (SUI) having lower urinary tract symptoms, participants were divided into two groups. The participants’ quality of life related to LUTS was assessed using the KHQ scale. The study group received e-PFMT along with behavioral therapy, while the control group received only behavioral therapy recommendations. Similar to our study, the results of this study reported that e-PFMT treatment conducted via video conferencing was a more effective protocol than behavioral therapy alone in improving LUTS symptom severity and associated quality of life [46].

In our study, when comparing the KHQ results of the groups before treatment, we observed that they were similarly affected in all subheadings related to symptom severity and quality of life, without any difference between them in terms of symptom severity. When the KHQ scale results were reapplied after treatment, we found that there was a difference between the AE and Control groups in the subheadings of general health, social limitation, emotional state, and symptom severity, an improvement in the AE group was observed due to application of highly effective treatment modality. When comparing the subheadings of role limitation, physical limitation, personal relationships, sleep, and energy level after treatment, some improvement was seen regarding these parameters in both groups, without any significant significant intergroup difference. We believe that this lack of difference is due to the fact that the symptomatic effects of these subheadings were already low in both groups prior to treatment. When the treatment effect size within the groups was examined after treatment, a high treatment effect size was found in the AE group for all subheadings compared to pre-treatment values. In the control group, improvement was observed after treatment compared to pre-treatment only in the subheadings of incontinence effect, role limitation, physical limitation, personal relationships, sleep, and energy level. Treatment effect sizes were found to be low in all subheadings except physical limitation compared to those in the AE group.

In our study, using KHQ scale assessments, we observed that the aerobic exercise program we implemented in addition to behavioral therapy in the AE group improved post-treatment LUTS symptoms and symptom severity in individuals with diabetes and LUTS, compared to the control group. This led to improvements in women’s relationships with their environment or their standing in society, in their circle with friends and neighbors, and thus in their social environment, and decrease the number of their ‘social limitations’ such as taking precautions as carrying a spare set of clothes or restricting intake of liquids (coffee, tea) due to frequent urination and fear of urinary incontinence associated with LUTS. The decrease in social limitations also explains the improvement in the “emotional state” subheading, which examines emotional states such as anxiety, irritability, and depression. As a result of all this, it has also positively affected their answers to the question “How would you describe your overall health?” and improvement has been seen in the “perceived overall health” subheading, and an improvement in quality of life has been found in the AE group compared to the Control group. In the section titled ‘Role limitation’, which examines participants’ performance at home, at work, and in daily life, we believe that another reason for the improvements observed at the same rate both in the AE and Control groups at the end of the study is that the behavioral modification recommendations given to both groups in the Behavioral Therapy application constitute a sufficient treatment approach with favorable outcomes. The reasons for improvement we put forward for the subheadings are supported by the literature [47, 48].

When we examined the BFLUTS results, we found that the AE and Control groups had similar symptomatic characteristics in the pre-treatment assessment in all subheadings that questioned storage functions, voiding functions, urinary incontinence, sexual life, and quality of life. In the post-treatment evaluation, we found a significant improvement in the AE group in the subheadings of storage functions and quality of life, as well as in the total BFLUTS score. In a study conducted by Kumsar et al. with 426 women with diabetes investigating the relationship between LUTS and sexual life, BFLUTS was used as the assessment scale. The average score for the sexual life subheading (1.37 ± 1.49) was similarly low compared to the averages of the groups in our study (AE group = 1.27 ± 1.16, Control group = 1.95 ± 1.53) [49]. LUTS is shown to be a factor that significantly and negatively affects sexual life and quality of life in women [39]. We believe that the results of Kumsar et al.‘s study and our study, contrary to the literature, suggest that women with diabetes with LUTS have low symptomatic impact on their sexual life which we correlated this phenomenon to various etiological factors. One of them is the relatively older women participating in these studies and the presence of individuals with inactive sexual lives in the study population due to various social reasons. Another reason is that the vast majority of participants answered “no” to the question, “Do you experience urinary incontinence during sexual intercourse?”

Our study found that women with diabetes had mild lower urinary tract symptoms when considering the maximum total score achievable for their total BFLUTS scores. This result is consistent with the results of the study conducted by Kumsar et al. on women with diabetes [49]. In contrast to the KHQ scale scores, which assessed quality of life and symptom severity, milder symptom severity according to the BFLUTS results is thought to be due to the fact that the population we studied had very few symptoms of voiding dysfunction, and urinary incontinence with little impact on sexual life, and the majority experiencing OAB-dry symptoms. Therefore, we can consider that the reason why these two scales, which are used for similar purposes such as assessing quality of life and symptom severity, produce different levels of LUTS outcomes when applied to the same participant group is that these scales evaluate LUTS symptoms under different headings. When examining the treatment status within the BFLUTS scale group, improvement was observed in all quality of life parameters except for the sexual life subheading in the AE exercise group after treatment, while improvement was only observed in the total score subheading in the Control group. With these results, we observed that AE therapy administered alongside behavioral therapy to women with diabetes with LUTS is a more effective treatment in improving quality of life and symptom severity of these patients.

## Limitations of the study

Although the exercises performed via video conferencing were supervised by a s, the need for physical contact to make adjustments during the exercises is a significant limitation. However, this limitation was minimized by providing face-to-face training on the exercises to be performed before starting the intervention. Furthermore, the fact that individuals with diabetes often turn to online exercise content due to ease of access, supports our preference for teleconferencing. However, our previous YouTube analysis showed that most of this content is not produced by health professionals and has low reliability [50]. Therefore, telerehabilitation programs conducted under the supervision of a physiotherapist offer a more reliable and effective option by overcoming the quality issues found in online content.

Another limitation is that urodynamic measurements are performed over a short period of time, and clinicians must interpret these short-term measurement results to generalize them to the patient’s overall bladder function. The other limitation is that during urodynamic measurements, the patient’s bladder filling and emptying activities occur in the presence of the clinician. This situation can affect the patient’s emotional state at that moment and, especially during measurements of intravesical pressure, can lead to misleading results. The clinician performing the measurements minimized this limitation by repeating measurements where controversial results were obtained.

## Conclusion

In conclusion, AE training given in conjunction with the behavioral treatment approach, which is recommended as first-line treatment in guidelines for women with DM and LUTS, was found to have a beneficial effect on bladder function, urinary system symptoms, and quality of life. Clinically, it is thought that aerobic exercise should be recommended early on in women with diabetes not only for glycemic control but also as a preventive strategy against the development of LUTS triggered by diabetes-related complications. In women with late-stage DM diagnosed with LUTS, we believe that referring them to a physiotherapist after informing them about the benefits of aerobic exercise, but before the severity of symptoms significantly affects their daily lives, would be a comprehensive and cost-effective approach to managing LUTS. In this context, we believe that AE training should be added to the guidelines used in the management of LUTS and the treatment programs implemented in clinics for individuals with DM. However, further studies are needed in which aerobic exercise training is planned and long-term follow-ups are conducted taking into account classification of BMI.

## Data Availability

The authors are available and ready to supply the data upon any requests through the corresponding author.

## Declarations

The study was conducted according to the principles of Helsinki Declaration.
